# Epidemiology of injuries among snowboarding athletes in the talent transfer program: A prospective cohort study of 39,880 athlete-exposures

**DOI:** 10.1371/journal.pone.0306787

**Published:** 2024-07-11

**Authors:** Feng Gao, Haiwei Li, Chen He, Yi Qian, Sen Guo, Zhihong Zhao, Yawei Gong, Yingqi Zhao, Xiaohan Zhang, Lei Li, Jingbin Zhou

**Affiliations:** 1 Department of Injury and Arthroscopy Surgery, National Institute of Sports Medicine, Beijing, China; 2 School of Physical Education, Shanxi Normal University, Taiyuan, China; 3 Department of Orthopedics, Beijing Second Hospital, Beijing, China; Ningbo University, CHINA

## Abstract

**Background:**

Talent transfer (TT) program is an appropriate approach to address the talent gap evident in specific sports activities, while little is known about the injury characteristics of snowboarding athletes involved in the TT program.

**Objective:**

To determine the epidemiology of injuries among snowboarders involved in the TT program.

**Methods:**

A total of 244 athletes who were not previously engaged in winter sports were selected for training in snowboarding that lasted for 109 days. The injuries and at-risk exposures (A-Es) data were recorded by physicians. Injury rates (IRs), incidence rate ratios (IRRs), and injury proportion ratios (IPRs) were calculated and compared by sex and age groups.

**Results:**

The overall and time loss (TL) IR were 32.4/1000 A-Es and 12.2/1000 A-Es respectively. The overall and non-time loss (NTL) IRR were higher for female athletes than for male athletes. Additionally, the overall IRR and TL-IRR for female athletes were higher in those athletes who aged ≤15 years old. Over 93% of TL injuries resulted in participation restriction time of ≤7 days (male athletes, 93.94%; female athletes, 94.10%). Trunk (28.43%), knee joints (21.33%), and hand/wrist (16.53%) were found as the common sites of injury in both female and male athletes. The most frequent type of injury was contusion (male athletes: 53.00%, female athletes: 59.10%) resulted from ground/apparatus contact (male athletes: 75.10%, female athletes: 75.20%).

**Conclusion:**

The risk injury among snowboarding athletes involved in the TT program during the first snow season training was found noticeable, especially for younger female athletes. The high incidence of ground/apparatus contact-related injuries suggested the necessity of specifically designed training programs and braces for snowboarding athletes involved in the TT program.

## Introduction

Snowboarding appeared in the 1970s and was first included in the Winter Olympic Games in 1998 in Nagano, Japan. Thereafter, snowboarding has become a popular sports activity worldwide [1]. By the time of the 2022 Beijing Olympics, snowboarding had grown into a mega-sport with 11 disciplines, ranking fourth in medal count behind speed skating, freestyle skiing, and cross-country skiing, as well as the traditional sports of alpine skiing and biathlon [2].

A talent transfer (TT) program encompasses the process of identifying individuals with particular sports skills, facilitating their transition to a new sports discipline [3]. This approach serves as an appropriate method to address talent shortages in specific sports activities [4]. TT athletes capitalize on the product of their aptitude, personal attributes, and prior investment and can be rapidly transitioned into a new sport, making this path time-effective in the pursuit of success in a new sport [5]. Therefore, identifying potential TT options can be very attractive for national governing bodies to increase the success rate of talent identification and development programs by recycling talent [4]. On July 31, 2015, Beijing and Zhangjiakou in China were jointly awarded the hosting rights for the 2022 Winter Olympics. This designation presents both challenges and opportunities for the development of winter sports in the People’s Republic of China. During the 2018 Winter Olympics, the Chinese delegation was comprised of 80 competitors in 12 sports. The team failed to qualify for the final round in two-thirds of the sports and was totally awarded only 9 medals. To enhance China’s performance in the Winter Olympic Games and promote the development of winter sports, the General Administration of Sport of China launched the snowboard TT program in August 2018.

The researches in TT have concentrated on the principal factors that contribute to the success of TT. Rea T and Lavallee D. found that support services through a formal program and informal program, similarities within the sport, and degree of success were factors that the athletes perceived as important for a successful transfer into another sport and being competitive within their new sport [6]. In their investigation into the effectiveness and underlying mechanisms of TT, Collins et al. identified the most prominent theme to be the psychosocial mechanism of TT [4]. In contrast to the anthropometric and performance variables that underpin current talent transfer initiatives, participants identified a range of psycho-behavioural and environmental factors as key to successful transfer, as reported by Macnamara Á et al [7]. In a study, Teunissen J. W. and colleagues proposed that gymnastics could serve as a potential donor sport for canoe/kayak. They further suggested that handball and tennis could facilitate the broad development of young canoe/kayak athletes [8]. Nevertheless, research on the characteristics of athletes’ sports injuries in TT is relatively scarce, yet it is of great interest to gain insight into the injuries of TT athletes in different sports.

The literature on snowboarding injuries primarily focused on amateur snowboarders. The injury incidence among amateur snowboarders ranged from 2.05 to 16 per 1000 snowboarder days [9–12]. Novices and those with lower skill levels are most susceptible to injury [13–15]. The majority of the injuries associated with snowboarding result from falling and they mainly occur in the upper limbs [16–18], in which wrist is recognized as the most common site [17,18]. Beginners, however, have exhibited to be affected by less severe injuries than more experienced snowboarders [19]. Fewer studies reported injury characteristics among professional snowboarders. The incidence of injuries during competition ranges from 1.3 to 6.4 per 1000 runs among competitive snowboarders at the national or world level. The injury pattern was different from amateur snowboarders, with fewer wrist injuries and more knee injuries [14–16].

Previous studies have examined the incidence and patterns of injuries among recreational and professional snowboarders, while the characteristics of injuries for snowboarding athletes within the TT program have not yet been reported. The present study aimed to determine the epidemiology of injuries among snowboarding athletes in the TT program during training and provide injury prevention strategies for snowboarders in the TT program.

## Methods

### Participants

This study used an observational cohort design. A total of 244 athletes, who were not previously engaged in winter sports, were selected by the TT program’s administrators to participate in snowboarding in September 2018, including 60 male participants (mean age, 15.05±2.10 years old) and 184 female participants (mean age, 15.02±1.90 years old). Participants’ previous disciplines are described in Table 1. From October to November 2018, these athletes received land-based training. They began to receive snow-based training during the first snow season at Jilin Ski Resort (Jilin, China) in December 2018. The protocol for this study received approval from the Ethics Committee of the China National Institute of Sports Medicine on 28/10/2018 (2018002). All the participants or their parents/guardians signed informed consent prior to the participation of the study.

**Table 1:** The number of athletes in different disciplines.

Rank	Discipline	Number (%)
1	Athletics	82(33.61%)
2	Wushu	65(26.64%)
3	Roller Skating	19(7.79%)
4	Skateboarding	19(7.79%)
5	Gymnastics	10(4.1%)
6	Diving	9(3.69%)
7	Taekwondo	8(3.28%)
8	Trampoline Gymnastics	7(2.87%)
9	Wrestling	5(2.05%)
10	Judo	4(1.64%)
11	Boxing	3(1.23%)
12	Weightlifting	2(0.82%)
13	Basketball	2(0.82%)
14	Dancing	2(0.82%)
15	Swimming	2(0.82%)
16	Football	2(0.82%)
17	Canoe Slalom	1(0.41%)
18	Archery	1(0.41%)
19	Tennis	1(0.41%)
Total	-	244(100%)

### Definitions

An injury was defined as an injury or illness occurring during the first snow-based training season that required assessment or treatment by team physicians. A time loss (TL) injury was defined as an injury that restricted participation for ≥24 h, and a non-time loss (NTL) injury was defined as an injury that restricted participation for <24 h [20]. In the present study, none of the participants engaged in any competitions, thus, athlete-exposures (A-Es) were defined as one athlete participating in one training session, in which he or she was exposed to the possibility of athletic injury, regardless of the time associated with his or her participation [21]. According to prior studies, the mechanism of injury was divided into contact with ground and apparatus, contactless, player contact, overuse, illness/infections, etc. [20].

### Data collection

The first snow season training was lasted for 109 days from December 1, 2018 to March 19, 2019. All the participants underwent basic technical training related to snowboarding and were not categorized into different events.

Injuries were diagnosed by qualified physicians who are licensed to practice in the field of sports medicine. The injury information was recorded by physicians within the injury report form, which included the time, position, affected site, diagnosis, and injury mechanism. When multiple injuries stemmed from a single incident, separate injury report forms were completed for each injury if they had distinct injury definitions, involved different body parts, or met both of these criteria. Duplicate injuries were otherwise removed [22]. All physicians received prior training in the data recording process and also documented information pertaining to injury conditions, the daily number of participating athletes, and the number of A-Es. Data related to injuries and A-Es were extracted from the injury report forms.

### Statistical analysis

Statistical analysis was performed using the SAS 6.0 software (SAS Institute Inc., Cary, NC, USA). The injury and A-Es data were reported by age and gender. The proportion of injured players and injury rates per 1000 A-Es with 95% CI were calculated, following established methodologies described in previous research [23]. Injury incidence was compared via incidence rate ratio (IRR). These effect estimates were computed with consideration of gender and age. In accordance with the international snowboard/freestyle/freeski competition rules (ICR, edition January 2023), participants were required to have attained their 15th birthday to be eligible for major snowboarding competitions. Therefore, participants were categorized into two groups: ≤15 years old and >15 years old at the inception of the cohort. In addition, injury distributions were assessed for the specific injured body part, diagnosis, and injury mechanism. Injury proportion ratio (IPR) was employed to compare injury distributions across gender and age groups. Statistically significant findings were defined as those with IRR and IPR, along with 95% CI, which did not encompass the value of 1.00 [24]. The following serves as an illustrative instance of an IPR, wherein the comparison concentrates on the proportion of injuries to the trunk between male and female athletes.


$$IPR=\frac{\left(\frac{\text{trunk}\;\text{injuries}\;\text{in}\;\text{male}\;\text{athletes}}{\text{all}\;\text{injuries}\;\text{in}\;\text{male}\;\text{athletes}}\right)}{\left(\frac{\text{trunk}\;\text{injuries}\;\text{in}\;\text{female}\;\text{athletes}}{\text{all}\;\text{injuries}\;\text{in}\;\text{female}\;\text{athletes}}\right)}$$

## Results

### Overall injury incidence and IRR

Overall, 87.7% (95% CI, 83.56–91.85) of participants experienced at least one injury during training. Besides, 62.49% (n = 808) of injuries were NTL injuries (Table 2). The majority of TL injuries (94.02%, n = 456) resulted in participation restriction time less than 7 days. The overall IRI was 32.4/1000 A-Es (95% CI, 30.7–34.2) for all athletes, and TL-IR was 12.2/1000 A-Es (95% CI, 11.1–13.2) (Table 3).

**Table 2:** Participants, A-Es, and injury count in snowboarding male and female athletes involved in the TT program.

Data		Male athletes			Female athletes			ALL
		≤15 yrs	>15 yrs	Total	≤15 yrs	>15 yrs	Total	
**Participants**		34	26	60	110	74	184	244
**A-Es**		6005	4917	10922	17207	11751	28958	39880
**Injuries**	All	175	138	313	622	358	980	1293
	TL injuries	82	50	132	256	97	353	485
	NTL injuries	93	88	181	366	261	627	808
**Injured** **Athletes**	All injuries	33	24	57	92	65	157	214
	TL injuries	28	18	46	75	42	117	163
	NTL injuries	5	6	11	17	23	40	51

**Table 3:** Injury rates in snowboarding male and female athletes involved in the TT program (per 1000 A-Es, 95%CI).

Injury rate	Male athletes			Female athletes			ALL
	≤15 yrs	>15 yrs	Total	≤15 yrs	>15 yrs	Total	
**All injuries**	29.1(24.9–33.4)	28.1(23.5–32.7)	28.7(25.5–31.8)	36.2(33.4–38.9)	30.5(27.4–33.6)	33.8(31.8–35.9)	32.4(30.7–34.2)
**TL injuries**	13.7(10.7–16.6)	10.2(7.4–13.0)	12.1(10.0–14.1)	14.9(13.1–16.7)	8.2(6.6–9.9)	12.2(10.9–13.5)	12.2(11.1–13.2)
**NTL injuries**	15.5(15.4–15.6)	17.9(17.8–18.0)	16.6(16.5–16.7)	21.3(21.2–21.3)	22.2(22.1–22.3)	21.7(21.6–21.7)	20.3(18.9–21.6)

#### Sex-based differences

Overall, 95% (95% CI, 86.08–98.96) of male athletes and 85.33% (95% CI, 79.38–90.11) of female athletes experienced at least one injury during training. Furthermore, 57.83% (n = 181) of injuries in male athletes and 63.98% (n = 627) of injuries in female athletes were NTL injuries (Table 1). Most of TL injuries (male athletes: 93.94%, n = 124; female athletes: 94.10%, n = 332) resulted in participation restriction time less than 7 days. The overall IR was 28.7/1000 A-Es (95%CI, 25.6–32.0) for male athletes and 33.8/1000 A-Es (95% CI, 31.8–36.0) for female athletes. The TL-IR was 12.1/1000 A-Es (95% CI, 10.1–14.3) for male athletes and 12.2/1000 A-Es (95% CI, 10.9–13.5) for female athletes (Table 2). The IRRs for all injuries (1.18; 95% CI, 1.04–1.34) and NTL injuries (1.31; 95%CI, 1.11–1.54) were higher in female athletes than those in male athletes.

#### Age-based differences

No differences were found in IR between male athletes who aged ≤15 years old and those who aged>15 years old for all, NTL, and TL injuries. The IRRs for all injuries (IRR, 1.19; 95% CI, 1.01–1.35) and TL injuries (IRR, 1.80; 95%CI, 1.43–2.27) in female athletes who aged ≤15 years old were higher than in those who aged >15 years old.

### Discipline-based differences

The injury count, A-Es, injured athletes, and IRs among TT snowboarding athletes in various disciplines are presented in the supplementary file. Notably, IRRs for all injuries (IRR: 1.52, 95% CI: 1.24–1.86) and NTL injuries (IRR: 1.60, 95% CI: 1.24–2.05) were significantly higher in Wushu compared with Skateboarding.

### Injury sites

In male athletes, the most commonly injury sites included trunk (28.43%, n = 89), followed by hands/wrists (14.06%, n = 44), and knee joints (13.10%, n = 41). In female athletes, the most common injury sites included knee joints (21.33%, n = 209), followed by trunk (20.71%, n = 203), and hands/wrists (16.53%, n = 162) (Table 4).

**Table 4:** Injury sites in snowboarding male and female athletes involved in the TT program.

Injury site	Male athletes			Female athletes		
	≤15 yrs	>15 yrs	Total	≤15 yrs	>15 yrs	Total
**Head/face**	10(5.71)	16(11.59)	26(8.31)	39(6.27)	14(3.91)	53(5.41)
**Neck**	12(6.86)	4(2.9)	16(5.11)	35(5.63)	28(7.82)	63(6.43)
**Shoulder**	11(6.29)	15(10.87)	26(8.31)	57(9.16)	33(9.22)	90(9.18)
**Arm/elbow**	18(10.29)	7(5.07)	25(7.99)	28(4.5)	19(5.31)	47(4.8)
**Hand/wrist**	20(11.43)	24(17.39)	44(14.06)	98(15.76)	64(17.88)	162(16.53)
**Trunk**	56(32)	33(23.91)	89(28.43)	133(21.38)	70(19.55)	203(20.71)
**Hip/groin**	5(2.86)	3(2.17)	8(2.56)	32(5.14)	10(2.79)	42(4.29)
**Upper leg**	7(4)	7(5.07)	14(4.47)	25(4.02)	6(1.68)	31(3.16)
**Knee**	22(12.57)	19(13.77)	41(13.1)	133(21.38)	76(21.23)	209(21.33)
**Lower leg**	4(2.29)	0(0)	4(1.28)	16(2.57)	14(3.91)	30(3.06)
**Ankle**	8(4.57)	7(5.07)	15(4.79)	18(2.89)	14(3.91)	32(3.27)
**Foot**	2(1.14)	3(2.17)	5(1.6)	8(1.29)	10(2.79)	18(1.84)
**Total**	175(100)	138(100)	313(100)	622(100)	358(100)	980(100)

The IPR for trunk injuries was higher in male athletes than that in female athletes (IPR = 1.37, 95% CI: 1.11–1.70), whereas the IPR for knee joint injuries was higher in female athletes than that in male athletes (IPR = 1.63, 95% CI: 1.19–2.22). No age-based differences were found for body parts injured in each gender.

### Diagnosis

The most prevalent injury types observed were contusion (male athletes: 53.00%, female athletes: 59.10%) and strain (male athletes: 17.60%, female athletes: 15.20%). Among male athletes, the IPR for contusions was higher in those who aged ≤15 years old compared with those who aged >15 years old (IPR = 1.32, 95%CI: 1.06–1.65). Additionally, the IPR related to concussion was higher in male athletes than in female athletes (IPR = 1.75, 95%CI: 1.01–3.02). Notably, male athletes who aged >15 years old had a higher IPR of concussion than male athletes who aged ≤15 years old (IPR = 2.75, 95%CI: 1.07–7.04). For female athletes, IPR related to contusions was higher in those who aged >15 years old than those who aged ≤15 years old (IPR = 1.15, 95%CI: 1.04–1.28) (Table 5).

**Table 5:** Diagnosis of common types of injuries in TT snowboarding male and female athletes.

Diagnosis	Male athletes			Female athletes		
	≤15 yrs	>15 yrs	Total	≤15 yrs	>15 yrs	Total
**Contusion**	104(59.4)	62(44.9)	166(53)	348(55.9)	231(64.5)	579(59.1)
**Fracture**	7(4)	1(0.7)	8(2.6)	10(1.6)	2(0.6)	12(1.2)
**Dislocation**	1(0.6)	3(2.2)	4(1.3)	1(0.2)	0(0)	1(0.1)
**Spasm**	1(0.6)	0(0)	1(0.3)	3(0.5)	4(1.1)	7(0.7)
**Strain**	31(17.7)	24(17.4)	55(17.6)	109(17.5)	40(11.2)	149(15.2)
**Concussion**	6(3.4)	13(9.4)	19(6.1)	27(4.3)	7(2)	34(3.5)
**Sprain**	8(4.6)	12(8.7)	20(6.4)	45(7.2)	29(8.1)	74(7.6)
**Laceration**	8(4.6)	4(2.9)	12(3.8)	11(1.8)	5(1.4)	16(1.6)
**Inflammation**	1(0.6)	1(0.7)	2(0.6)	0(0)	4(1.1)	4(0.4)
**Heat event**	0(0)	0(0)	0(0)	1(0.2)	0(0)	1(0.1)
**Others**	8(4.6)	18(13)	26(8.4)	67(10.8)	36(10.1)	103(10.6)
**Total**	175(100)	138(100)	313(100)	622(100)	358(100)	980(100)

### Mechanism of injury

In both male and female athletes, the most common mechanism of injury was contact with the ground and apparatus (male athletes: 75.10%, n = 235; female athletes: 75.20%, n = 737), followed by contactless mechanism (male athletes: 14.10%, n = 44; female athletes: 14.10%, n = 138). (Table 6) No gender-based differences related to the mechanism of injury were found. In male athletes, however, the IPR due to overuse was higher in those who aged >15 years old than in those who aged ≤ 15 years old (IPR = 6.34, 95%CI: 1.41–28.47).

**Table 6:** Mechanism of injury in TT snowboarding male and female athletes.

Mechanism of injury	Male athletes			Female athletes		
	≤ 15 yrs	>15 yrs	Total	≤15 yrs	>15 yrs	Total
**Contact with ground and apparatus**	139(79.4)	96(69.6)	235(75.1)	453(72.8)	284(79.3)	737(75.2)
**Contactless**	24(13.7)	20(14.5)	44(14.1)	102(16.4)	36(10.1)	138(14.1)
**Player contact**	8(4.6)	8(5.8)	16(5.1)	19(3.1)	11(3.1)	30(3.1)
**Overuse**	2(1.1)	10(7.2)	12(3.8)	35(5.6)	19(5.3)	54(5.5)
**Illness/Infection**	0(0)	1(0.7)	1(0.3)	0(0)	1(0.3)	1(0.1)
**Others**	2(1.1)	3(2.2)	5(1.6)	13(2.1)	7(2)	20(2)
**Total**	175(100)	138(100)	313(100)	622(100)	358(100)	980(100)

## Discussion

In the present study, it was found that snowboarding athletes in the TT program were at a high risk of injury during the first snow season, especially younger female athletes. However, most of the injuries were minor, resulting in minimal or no training time loss. Trunk, knee joints, and hand/wrist were found as the common sites of injury in both female and male athletes. The most frequent type of injury was contusion resulted from ground/apparatus contact.

### IRs for snowboarding athletes involved in the TT program

Snowboarding is considered as a sport with a high risk of injury [14,25]. In the present study, 95% of male athletes and 85.33% of female athletes suffered from at least one injury, which indicated a high risk of injury among snowboarding athletes involved in the TT program. Among snowboarding amateurs who reported to have sustained injuries, about 36–58% [10,12,13] were novices. As a result, it can be inferred that the high risk of injury noted in the present study could be attributed to the fact that participants were novices. In addition, the TT program has progressed at a rapid pace with the involvement of participants who received training 2 or 3 sessions a day, which might contribute to the higher risk of injury.

Previous studies have suggested that the risk of injury is higher in male snowboarding athletes than that in female athletes [26]. This could be attributed to the fact that male athletes mainly opt for competition events that demand advanced skill levels. However, in the present study, both male and female athletes were involved in the same level of training, eliminating this as an influential factor. The present study demonstrated that IRs for all injuries and NTL injuries were higher in female athletes than those in male athletes. One possible explanation could be the higher proportion (45.1%) of younger female athletes participating in the TT program, and performance-related fitness of underage female athletes was significantly lower than that of adults.

Children and adolescents represent a demographic highly susceptible to snowboarding injuries, with the associated risk diminishing as athletes mature [27,28]. The findings of the present study partially support the notion that snowboarders in the younger age group face the highest risk of injury, particularly among female athletes in the TT program.

### Injury severity and injury sites among snowboarding athletes involved in the TT program

TL from training or competition due to injuries [29] serves as a metric to assess injury severity. It was attempted to categorize injury severity into five levels: slight (no absence), minimal (1–3 days), mild (4–7 days), moderate (8–28 days), and severe (>28 days) based on TL duration [30,31]. Notably, only 6.06% of male athletes and 5.90% of female athletes in the present study experienced moderate-to-severe TL injuries. This relatively lower severity of injuries may be attributed to the lower skill levels of the athletes in this study. Ogawa et al. [15] demonstrated that injury severity tends to increase with the athlete’s higher skill levels. In the present study, athletes’ low skills may explain the relatively low severity of injuries.

Upper limb injuries, particularly in the wrists and arms [14,32,33], were frequently reported [9,18,28,34,35], along with head, face [26,36], and knee joints [10,37]. In the present study, when combining shoulder, upper arm/elbow, and hand/wrist injuries, upper limb injuries accounted for 30.36% in male athletes and 30.51% in female athletes, aligning with previously reported findings [9,14,18,28,32–35]. Conversely, trunk injuries were prevalent in male athletes, while knee joint injuries were common in female athletes. Injuries to the trunk and hand/wrist seem to be associated with falls involving contact with the ground or apparatus, whereas knee joint injuries are primarily non-contact, mainly arising from a loss of control. The higher risk of knee joint injuries in female snowboarders may be attributed to gender-specific knee stability concerns [38].

### Diagnosis and mechanism of injury

It has been reported that fractures constitute the most frequent snowboarding injury, with incidence ranging from 31% to 39% [14,36]. However, other studies have identified contusion as the predominant diagnosis, with the incidence rate of 31–40.5% [28,32]. The present study aligns with the latter findings, revealing contusion and strain as the most frequent injury diagnoses in both male and female athletes. Notably, the incidence of fractures in this study was notably lower in male athletes (2.6%) and in female athletes (1.2%) compared with previous reports. This divergence could be attributed to athletes’ prior sports training experience within the TT program. Notably, the incidence of concussion in male athletes who aged over 15 years old was significantly higher than in those under 15 years old, which is consistent with previous outcomes [39,40].

Regarding injury mechanisms, earlier studies have emphasized falling, jumping, and collisions as primary causes of snowboarding injuries [15,32,36]. In the present study, the most common injury mechanism was contact with the ground and apparatus, followed by contactless mechanism. Dickson et al. [41] found that fall-induced injuries accounted for 79.9%, while loss of control contributed to 10.5% of wrist injuries in snowboarders, which was comparable with the present study. However, few studies have addressed contactless injuries among low-skilled snowboarders [17,41]. Additionally, injuries attributed to overuse were more prevalent in male athletes who aged over 15 years old compared with those under 15 years old, which could be related to their prior training exposure and chronic injuries.

## Limitations

The present study has several limitations that should be pointed out. Firstly, a relatively small cohort of 244 snowboarding athletes, who underwent training in the TT program, could be examined. Consequently, the generalizability of findings may be limited to this specific program. Secondly, no specific injury diagnoses were presented for different anatomical sites. For instance, in cases of wrist injuries, distal radius fractures were not distinguished from medial collateral ligament tears. Moreover, athletes in this study exclusively participated in snow-based training, precluding an evaluation of injury risks associated with competitive events. Additionally, injury rates could only be compared with those of studies employing the same injury exposure estimation method. Lastly, the TT program included a total of 224 athletes across 19 disciplines, with 15 of those disciplines having fewer than 10 athletes each. Consequently, analyzing the impact of athletes’ prior disciplines on injury risk could be challenging.

## Conclusions

In conclusion, this study revealed that snowboarding athletes involved in the TT program could face a substantial risk of injury during their initial snow season training, and younger female athletes were found to be particularly vulnerable. The analysis of injury mechanisms underscored the importance of specially designed land-based training programs aimed at preventing collisions with equipment or falling and mitigating contactless injuries arising from loss of control. Equally crucial for injury prevention in the TT program is the use of braces for the wrists, elbows, and trunk (particularly concentrating on the gluteal region).

## Supporting information

S1 File TABLE 1. is about the different disciplines of participants, A-Es, and injury counts in TT snowboarding athletes. TABLE 2. is about injury rates of different disciplines of TT snowboarding athletes (per 1000 A-Es, 95%CI).
(DOCX)
