# Supplementary material for: Epidemiology of injuries among snowboarding athletes in the talent transfer program: A prospective cohort study of 39,880 athlete-exposures
Source: PLoS One. 2024 Jul 11;19(7):e0306787. doi: 10.1371/journal.pone.0306787 (PMC11239072; doi:10.1371/journal.pone.0306787)
Supplement: S1 File — TABLE 1. is about the different disciplines of participants, A-Es, and injury counts in TT snowboarding athletes. TABLE 2. is about injury rates of different disciplines of TT snowboarding athletes (per 1000 A-Es, 95%CI). (DOCX) [file pone.0306787.s001.docx]

Supplemental file

TABLE 1. Different disciplines of participants, A-Es, and injury counts in TT snowboarding athletes

| Disciplines | N | A-Es | All Injuries | TL injuries | NTL injuries | Injured athletes |
| --- | --- | --- | --- | --- | --- | --- |
| Athletics | 82 | 13221 | 415 | 148 | 267 | 68 |
| Wushu | 65 | 10885 | 297 | 106 | 191 | 57 |
| Roller Skating | 19 | 3728 | 116 | 49 | 67 | 18 |
| Skateboarding | 19 | 3076 | 129 | 42 | 87 | 19 |
| Gymnastics | 10 | 1654 | 75 | 33 | 42 | 8 |
| Diving | 9 | 1330 | 65 | 31 | 34 | 9 |
| Taekwondo | 8 | 1422 | 61 | 23 | 38 | 8 |
| Trampoline Gymnastics | 7 | 918 | 30 | 14 | 16 | 7 |
| Wrestling | 5 | 686 | 14 | 10 | 4 | 3 |
| Judo | 4 | 556 | 17 | 6 | 11 | 2 |
| Boxing | 3 | 513 | 26 | 2 | 24 | 3 |
| Weightlifting | 2 | 184 | 2 | 0 | 2 | 1 |
| Basketball | 2 | 317 | 15 | 5 | 10 | 2 |
| Dancing | 2 | 269 | 10 | 5 | 5 | 2 |
| Swimming | 2 | 259 | 2 | 1 | 1 | 1 |
| Football | 2 | 290 | 7 | 4 | 3 | 2 |
| Canoe Slalom | 1 | 209 | 2 | 0 | 2 | 1 |
| Archery | 1 | 210 | 4 | 1 | 3 | 1 |
| Tennis | 1 | 153 | 6 | 5 | 1 | 1 |

TABLE 2. Injury rates of different disciplines of TT snowboarding athletes (per 1000 A-Es, 95%CI)

| Disciplines | **N** | Injury rates | | |
| --- | --- | --- | --- | --- |
|  |  | All Injuries | TL injuries | NTL injuries |
| Athletics | 82 | 31.4(28.5-34.6) | 11.2(9.5-13.2) | 20.2(17.9-22.8) |
| Wushu | 65 | 27.3(24.4-30.6) | 9.7(8.0-11.8) | 17.5(15.2-20.2) |
| Roller Skating | 19 | 31.1(25.9-37.3) | 13.1(9.8-17.4) | 18(14.1-22.9) |
| Skateboarding | 19 | 41.9(35.2-49.7) | 13.7(10.0-18.6) | 28.3(22.9-3.50) |
| Gymnastics | 10 | 45.3(36.0-56.8) | 20.0(14.0-28.3) | 25.4(18.6-34.5) |
| Diving | 9 | 48.9(38.2-62.3) | 23.3(16.1-33.3) | 25.6(18.1-36.0) |
| Taekwondo | 8 | 42.9(33.2-55.1) | 16.2(10.5-24.6) | 26.7(19.2-36.8) |
| Trampoline Gymnastics | 7 | 32.7(22.6-46.9) | 15.3(8.7-26.2) | 17.4(10.3-28.8) |
| Wrestling | 5 | 20.4(11.6-34.9) | 14.6(7.4-27.6) | 5.8(1.9-15.9) |
| Judo | 4 | - | - | - |
| Boxing | 3 | - | - | - |
| Weightlifting | 2 | - | - | - |
| Basketball | 2 | - | - | - |
| Dancing | 2 | - | - | - |
| Swimming | 2 | - | - | - |
| Football | 2 | - | - | - |
| Canoe Slalom | 1 | - | - | - |
| Archery | 1 | - | - | - |
| Tennis | 1 | - | - | - |

- 1、The calculation of injury rates is no longer conducted for players with fewer than five individuals.
